# Effects of Melatonin, GM-CSF, IGF-1, and LIF in Culture Media on Embryonic Development: Potential Benefits of Individualization

**DOI:** 10.3390/ijms25020751

**Published:** 2024-01-06

**Authors:** Jung-Won Choi, Sung-Woo Kim, Hee-Sun Kim, Moon-Joo Kang, Sung-Ah Kim, Ji-Yeon Han, Hoon Kim, Seung-Yup Ku

**Affiliations:** 1 Laboratory of In Vitro Fertilization, Department of Obstetrics and Gynecology, Seoul National University Hospital, Seoul 03080, Republic of Korea; cjungwon0702@gmail.com (J.-W.C.); 20555@snuh.org (H.-S.K.); 20717@snuh.org (M.-J.K.); 21358@snuh.org (S.-A.K.); 2 Department of Obstetrics and Gynecology, Seoul National University Hospital, Seoul 03080, Republic of Korea; sungwookim@snu.ac.kr (S.-W.K.); garda747@gmail.com (J.-Y.H.); obgyhoon@gmail.com (H.K.); 3 Department of Obstetrics and Gynecology, Seoul National University College of Medicine, Seoul 03080, Republic of Korea; 4 Institute of Reproductive Medicine and Population, Medical Research Center, Seoul National University, Seoul 03080, Republic of Korea

**Keywords:** supplements, culture media, embryonic development, individualization, in vitro fertilization

## Abstract

The implantation of good-quality embryos to the receptive endometrium is essential for successful live birth through in vitro fertilization (IVF). The higher the quality of embryos, the higher the live birth rate per cycle, and so efforts have been made to obtain as many high-quality embryos as possible after fertilization. In addition to an effective controlled ovarian stimulation process to obtain high-quality embryos, the composition of the embryo culture medium in direct contact with embryos in vitro is also important. During embryonic development, under the control of female sex hormones, the fallopian tubes and endometrium create a microenvironment that supplies the nutrients and substances necessary for embryos at each stage. During this process, the development of the embryo is finely regulated by signaling molecules, such as growth factors and cytokines secreted from the epithelial cells of the fallopian tube and uterine endometrium. The development of embryo culture media has continued since the first successful human birth through IVF in 1978. However, there are still limitations to mimicking a microenvironment similar to the reproductive organs of women suitable for embryo development in vitro. Efforts have been made to overcome the harsh in vitro culture environment and obtain high-quality embryos by adding various supplements, such as antioxidants and growth factors, to the embryo culture medium. Recently, there has been an increase in the number of studies on the effect of supplementation in different clinical situations such as old age, recurrent implantation failure (RIF), and unexplained infertility; in addition, anticipation of the potential benefits from individuation is rising. This article reviews the effects of representative supplements in culture media on embryo development.

## 1. Introduction

Since the birth of the first baby conceived through in vitro fertilization (IVF) in the late 1970s, IVF has continuously developed as an important field of assisted reproductive technology (ART), providing numerous infertile couples with the opportunity to bear children. Among its pivotal components of IVF, the quality of embryos plays a crucial role in determining the success of IVF procedures. The transfer of good-quality embryos has been shown to result in higher rates of clinical pregnancy and live birth [1]. The transfer of poor-quality embryos was associated with an increase in the miscarriage rate, which ultimately led to a decrease in the live birth rate [2,3].

Embryo culture media constitute a vital aspect of the IVF process, providing a controlled environment for embryo development outside the maternal uterus [4]. In vitro cultured embryos often show delayed or abnormal development during the cell division phase, suggesting a lack of essential factors that are naturally present within the female reproductive tract. Researchers have investigated supplementing culture media with various bioactive molecules due to the increased prevalence of infertility stemming from factors like advanced age, recurrent failure (RIF), and unexplained infertility [5,6,7,8,9].

This review aims to summarize the effects of various supplements in culture media on embryo development by comprehensively reviewing the existing literature. The study population, experimental methodologies, and outcomes from in vitro and in vivo studies were examined, focusing on the impact of supplements on various aspects of embryo development, including embryo quality, blastocyst formation, implantation potential, and pregnancy outcomes. Through a thorough evaluation of existing evidence, this review endeavors to consolidate our understanding of the potential advantages of incorporating these supplements into embryo culture media. Ultimately, a comprehensive assessment of their effects will aid in optimizing the culture conditions for improving the outcomes of IVF procedures, offering new avenues to enhance the success rates and overall reproductive health of individuals undergoing ART.

## 2. Effect of Supplements in IVF Culture Media on Embryonic Development

Since the first human IVF baby was born on 25 July 1978, there have been great advancements in clinical human embryo culture conditions [10,11]. Culture conditions that affect embryos cultured in vitro are crucial factors related to pregnancy outcomes [12,13,14,15,16]. Among air quality, culture medium, incubator type, temperature, and pH, the composition of the culture medium holds paramount importance, as it is in direct contact with the embryo and engages with it via paracrine and autocrine mechanisms [17].

Research on the impact of embryo culture media on embryos in ART cycles has investigated parameters such as embryo quality, pregnancy outcomes, and neonatal outcomes. In a comparative study of two culture media, GⅢ (Vitrolife, Gothenburg, Sweden) media exhibited higher quality and more viable embryos, and correlated with heightened rates of implantation and pregnancy success in frozen–thawed cycles compared to G1.2-G2.2 (Vitrolife, Gothenburg, Sweden) media [18]. Similarly, significant differences were observed between the G5 culture medium and HTF culture medium in implantation rates, clinical pregnancy rate, and birth weight [19]. A UK study reported the impact of eight different culture media types—Cook sequential, Irvine Single-step, LifeGlobal Single-step, Sage sequential, and Vitrolife sequential, among others—on the live birth rate [20]. Conversely, there are studies indicating no difference in miscarriage rate, live birth rate, and birth weights among different culture media [21,22]. Efforts have been made to enhance culture media for in vitro embryos, recognizing the pivotal role of medium composition compared to the in vivo environment’s trophic support. Therefore, four different culture media most commonly used for embryo development, namely, G-TL (Vitrolife, Gothenburg, Sweden), 1-Step (Origio, Måløv, Denmark), Global-Total (LifeGlobal, Guilford, CT, USA), and CSC (Irvine Scientific, Santa Ana, CA, USA), were investigated with a focus on their composition in terms of components critical for embryo growth (Table 1) [23,24,25,26,27,28,29,30]. The primary energy substrates for preimplantation embryos include pyruvate, lactate, and glucose, which were present in all four media, albeit at varying concentrations. Amino acid compositions and ionic electrolyte levels also varied significantly among the four media [31,32]. The amino acid and electrolyte levels should be maintained in the embryo culture media for synthesis of protein, cell signaling, metabolism, cell balance, pH and membrane stability [33,34].

**Figure 1:**
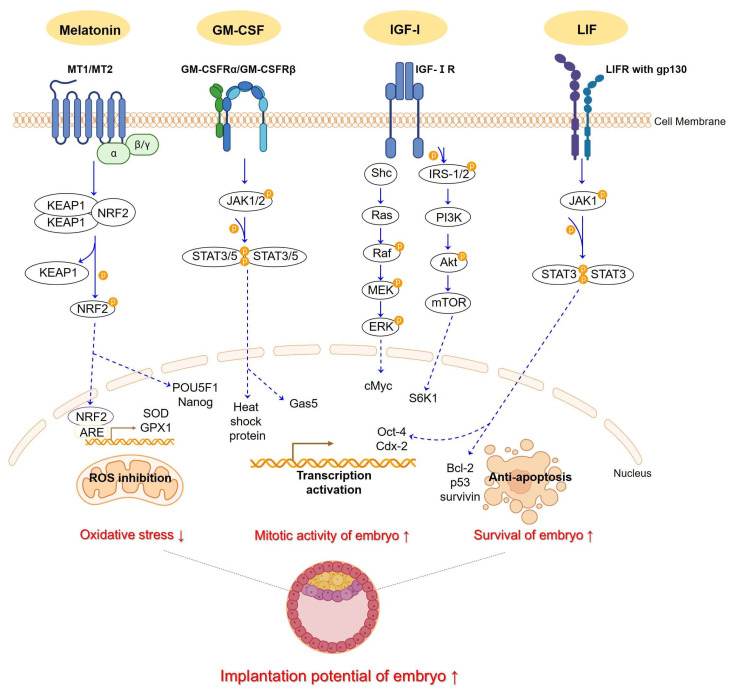
An illustrative schematic depicts how melatonin, GM-CSF, IGF-1, and LIF pathways function in embryo culture media, showing their expected effects. Melatonin binding to MT1,2 receptors trigger the KEAP1/NRF2 complex formation. Protein kinases aid NRF2 dissociation, enabling its translocation. NRF2, post-translocation, binds ARE, initiating transcription of *SOD* and *GPX1* and activating *POU5F1* and *Nanog*. GM-CSF binds to its receptors, GM-CSFRα/β. Upon binding, the receptors undergo autophosphorylation, activating the JAK1,2/STAT3,5 pathway. Once STATs are activated, they translocate and form dimer to suppress target genes, including *heat shock protein* and *Gas5*. IGF-Ⅰ interacts with its receptors, IGF-ⅠR. Upon binding, the receptor autophosphorylates and recruits IRS-1/2 and Shc. This activates the PI3K/Akt/mTOR pathway and *S6K1* gene. Simultaneously, Shc recruitments activate the Ras/Raf/MEK/ERK pathway and *cMyc* gene. LIF binds to its receptor, LIFR, along with gp130. Upon binding, it promotes receptor heterodimerization, triggering the activation of the JAK1,2/STAT3 pathway. This activation results in the regulation of apoptosis-related genes, such as *Bcl-2*, *p53*, and *survivin*. It also activates the crucial transcription genes, *Oct-4* and *Cdx-2*. These supplementations ultimately have the potential to reduce oxidative stress in the embryo and elevate mitotic activity and survival, thus presenting promise for successful implantation. The dotted line indicates translocation in each pathway. This Figure was created using PPT Drawing Toolkits-BIOLOGY Bundle from Motifolio, Inc, and created with BioRender.com.

Signal pathways regulate transcription factors that the embryo encounters, potentially altering its development competence [35,36,37,38,39,40]. Supplements in embryo culture media include hormones, growth hormones, growth factors, and cytokines. In this review, we selected the most frequently studied supplementations for each class of compound for investigation. Hence, we have chosen four molecules—melatonin, GM-CSF, IGF-Ⅰ, and LIF—and conducted a review regarding their molecular signal pathways (Figure 1) and potential impact on pre-implantation embryo development and, if available, pregnancy outcomes.

### 2.1. N-Acetyl-5-Methoxytryptamine (Melatonin)

Melatonin, derived from tryptophan and originating in the pineal gland, exhibits a circadian rhythm and is primarily secreted during the dark hours of the night [41,42]. It is predominantly synthesized within the mitochondria, and can easily pass through the cell membrane due to its amphiphilic nature [43,44]. Melatonin acts as an activator of antioxidants and regulator of inflammation, effectively scavenging free radicals in organisms [45,46,47]. Melatonin could increase the activity of other antioxidant enzymes, such as glutathione and superoxide dismutase (SOD), and is an inhibitor of pro-oxidant enzymes. In mammals, the melatonin receptor functions as a G protein-coupled protein (GPCR) with two known types: Mt1 and Mt2 [48]. The elevated expression of proteins (NRF2 and KEAP1) after melatonin treatment offers evidence that melatonin effectively protects *IVF*-derived embryos from oxidative stress through the Nrf2/ARE signaling pathway [49]. Melatonin exhibits antioxidant properties by regulating the expression of *NFE2L2*, *SOD1, GPX1*, and *GPX4* genes. Melatonin also activates essential genes, such as *ErbB1*, *ErbB4*, *GJA1*, *POU5F1*, *Nanog*, and *vascular endothelial growth factor* (VEGF) and VEGF *type 1 receptor* (VEGF-R1), which are involved in embryo implantation, blastocyst growth, and angiogenesis (Figure 1) [50,51,52].

During embryo development, oxygen is used in three pathways to produce adenosine triphosphate, fulfilling energy needs and converting some of it into reactive oxygen species (ROS) [53,54]. ROS, including the superoxide anion (O_2_^−^), hydrogen peroxide (H_2_O_2_), and the hydroxyl radical (•OH), can accumulate inside gametes and embryos due to oocyte activation, cleavage, transcription regulation, and hatching to implantation and exogenous factors during ART [55,56,57,58,59,60,61,62]. Optimal ROS levels sustain cellular metabolism and embryonic development, while excessive ROS may adversely impact embryo morphokinetics, gene expression, and survival [63,64]. Cells have antioxidant mechanisms to defend against these ROS [65]. There are two categories of antioxidant systems: enzymes like SOD, catalase, and glutathione peroxidase (GSH); and non-enzyme types like vitamins, polyphenols, carotenoids, and minerals. During the in vivo preimplantation stage of embryo development, embryos protect themselves with oxygen scavengers that are present in the reproductive tract, such as vitamins, pyruvate, GSH, SOD, and cysteamine [66]. However, embryos cultured in vitro rely solely on their intrinsic antioxidant pathways to rescue ROS without support from the maternal environment. Therefore, although there are still pros and cons, the need for research to reduce ROS by adding antioxidants to the embryo culture medium has emerged.

Among the studies investigating the effects of melatonin, half were retrospective randomized controlled trial (RCT)s on humans and the rest were studies on animals (Table 2). Most recently, a human clinical study using 10^−7^ M of melatonin in the embryo culture medium investigated the blastocyst development rate and gene expression [67]. The addition of melatonin to the embryo culture medium improved the rate of high-quality day 3 embryos (29.6% (Melatonin) vs. 19.5% (Control), *p* = 0.0123) in patients with repeated poor-quality-embryos, although no significant difference was observed in the clinical pregnancy rate. In addition, melatonin enhanced the blastocyst development rate (42.25% (Melatonin) vs. 26.38% (Control), *p* < 0.05) in the patients with frozen–thawed cycles. The ROS levels did not differ significantly between the two groups, but only the *CAT* gene exhibited a significant increase in the cultured blastocysts among the anti-apoptosis and antioxidant genes (1.68 ± 0.43 (Melatonin) vs. 1.00 ± 0.09 (Control), *p* < 0.05). This research indicates that melatonin supplementation could benefit repeated poor-quality embryos as well as frozen–warmed embryos in terms of the preimplantation embryo development rate and quality. It suggests that further research is needed using a large-scale sample and focusing on implantation outcomes.

Another human study investigated the embryonic development rate, implantation rate, and live birth rate based on the presence or absence of melatonin supplementations; this study used 140 patients who underwent at least one failed repeated IVF/ICSI cycle [68]. The melatonin group showed a significant increase in fertilization rate (87.7% (Melatonin) vs. 83.6% (Control), *p* < 0.01), cleavage development rate (94.1% (Melatonin) vs. 90.5% (Control), *p* < 0.01), high-quality embryo rate (58.3% (Melatonin) vs. 43.8% (Control), *p* < 0.0001), and high-quality blastocyst development rate (43.4% (Melatonin) vs. 22.9% (Control), *p* < 0.0001). When the vitrified/warmed blastocysts were transferred, the implantation rate (65.6% (Melatonin) vs. 9.7% (Control), *p* < 0.0001) and the clinical pregnancy rate (40.0% (Melatonin) vs. 11.7% (Control), *p* < 0.0001) were significantly higher in the melatonin group. Notably, the melatonin group included two women who delivered two healthy individual newborns. This demonstrates that melatonin-containing embryo culture media could increase both preimplantation embryo development and clinical outcomes; this finding particularly stands out with regard to the implantation rate and live births in patients with repeated failed IVF/ICSI cycles.

Another RCT was carried out with polycystic ovary syndrome (PCOS) patients with in vitro maturation (IVM)-IVF cycles; in this RCT, 10 μmol/L of melatonin was added to the culture medium [69]. PCOS patients showed significantly higher implantation rates in the melatonin-supplemented group both when using non-stimulation protocols (22.6% (Melatonin) vs. 11.6% (Control), *p* < 0.05) and human chorionic gonadotropin (hCG) priming protocols (26.4% (Melatonin) vs. 12.7% (Control), *p* < 0.05). However, there were no differences in embryo development competency and ongoing pregnancy rate. This study showed that melatonin could bring beneficial effects to PCOS patients’ IVM and implantation competency.

The most recent study with in vivo-derived and in vitro-derived porcine embryo examined the effects of the sequential addition of melatonin to the IVM, IVF, and culture medium by development competence [70]. Melatonin accelerated embryo development (*p* < 0.05), showing a significant increase in the cleavage development rate (*p* < 0.001) while indicating no notable difference in the blastocyst rate. Additionally, intracellular glutathione levels, (~40% increase, *p* < 0.03) showed a significant increase, while the reactive oxygen species level showed a significant decrease (~26% decrease, *p* < 0.01) in the melatonin group. Ultimately, this study shows that exogenous melatonin addition could increase the cleavage development rate but not the blastocyst development rate or its cell number. In addition, the study suggests that melatonin supplements in culture media function like antioxidants; this finding was supported by a decrease in the intracellular levels of oxygen free radicals and deoxyribo nucleic acid (DNA) damage in in vitro porcine embryos.

The animal study analyzed the developmental rate and gene expression using 10^−7^ M melatonin, investigating its impact on in vitro vitrified/warmed bovine embryos [71]. The melatonin group exhibited a higher cleavage development rate (87.78 ± 1.02% (Melatonin) vs. 82.50 ± 1.12% (Control), *p* < 0.05) and blastocyst development rate at day 7 (38.33 ± 2.21% (Melatonin) vs. 26.67 ± 1.05% (Control), *p* < 0.05) compared to the control group. Furthermore, melatonin improved the re-expansion of vitrified/warmed blastocysts and development-related gene expression levels. In summary, this finding indicates that melatonin enhances the progression of in vitro embryo development and the cryotolerance of blastocysts. In addition, vitrified/warmed blastocysts demonstrated increased re-expansion rates, potentially mediated by the regulation of gene expression, including *DNMT3A*, *OCC*, *CDH1*, and *AOP3*.

Using 10^−7^ M of melatonin, the same author investigated the effects of melatonin on embryo development competence and its impact on conception and birth outcomes [72]. There was a significant difference in the cleavage development rate (92.0 ± 5.68% (Melatonin) vs. 89.3 ± 4.17% (Control), *p* < 0.05) and the blastocyst development rate (80.3 ± 4.57% (Melatonin) vs. 64.6 ± 6.04% (Control), *p* < 0.05) in the melatonin group. Using a recipient mouse with transferred embryos, the implantation rate (95.0 ± 3.42% (Melatonin) vs. 67.8 ± 5.03% (Control), *p* < 0.01), litter size (4.1 ± 0.37 pups/litter (Melatonin) vs. 2.7 ± 0.42 pups/litter (Control), *p* < 0.05), and the survival rate (96.8 ± 2.15% (Melatonin) vs. 81.2 ± 4.36 (Control), *p* < 0.05) were increased in the melatonin group while the body weight of the offspring was comparable between the two groups. Additionally, gene expressions associated with antioxidant and apoptotic factors were regulated differently in embryos at each developmental stages in the two groups. The author concluded that a higher pregnancy rate could be linked to the improved blastocyst following melatonin treatment, as the melatonin group showed an increase in the cell number of blastocysts and decreases in the apoptotic rate. Similarly, improved pregnancy quality in recipients led to the birth of heal their offspring, resulting in increased postnatal survival.

Overall, current studies support the finding that melatonin may enhance preimplantation embryonic development [67,68,70,71,72] and the success rate of implantation [68,72], as indicated by human clinical studies and animal research. Furthermore, some studies indicate that melatonin could result in a reduction in ROS during embryo development, and this may lead to a decrease in DNA damage [70,72,73]. While human studies have shown consistent results, the dosage of melatonin varied within the same species, highlighting the need for further research to standardize its dosage before clinical application. Additionally, the exact mechanism by which melatonin affects embryos in in vitro culture remains unclear. Since melatonin is not a major essential factor for pregnancy, there is a greater need to determine whether its addition to culture media has any negative impact on fetus and perinatal outcomes. Therefore, further investigation of the downstream signaling pathways is necessary to gain a precise understanding of how melatonin influences embryo development. Additionally, melatonin operates in conjunction with other molecules rather than in isolation, potentially forming intricate interactions. Thus, a comprehensive approach encompassing diverse perspectives is imperative for its thorough research and application. Melatonin shows promise as a beneficial addition to embryo culture media, particularly when substantiated by studies featuring a larger sample size, multicenter involvement, and comprehensive long-term follow-ups, including birth outcomes.

**Table 2:** Effects of melatonin in culture media on embryonic development and pregnancy outcomes.

Year	Model	Study Type	Dose of Melatonin	Timing of Intervention	Cleavage Development Rate	Blastocyst Development Rate	Embryo Grade	Implantation Rate	Live Birth Rate	Ref.
2022	Human (unexplained infertility)	RCT	10^−7^ M	COC	-	-	↑ day 3	-	-	[67]
				Day 3 embryo after vitrified/warmed	-	↑	↑ day 5	-	-	
2022	Human (RIF)	RCT	10^−9^ M	COC or oocyte	↑	↑	↑, ↑ day 3, day 5	↑	↑	[68]
2013	Human (PCOS)	RCT	10^−6^ M	Immature COC surrounded by compact cumulus cells	→	-	→ day 3	↑	-	[69]
2022	Porcine	animal	10^−9^ M	Presumed zygote	↑	→	-	-	-	[70]
2014	Bovine	animal	10^−7^ M	Zygote	↑	↑	-	-	-	[71]
2013	Murine	animal	10^−7^ M	Zygote	↑	↑	-	-	↑	[72]

-: not analyzed; →: non-significance; ↑: significantly increased.

### 2.2. Granulocyte-Macrophage Colony-Stimulating Factor (GM-CSF)

GM-CSF, also known as CSF2, is the most well-studied cytokine among the CSF group of cytokines. GM-CSF has specific receptors that are comprised a cytokine-specific α-chain (GM-Rα) and b-chain (GM-Rβ) [74,75]. The main target cells of GM-CSF are immune cells, myeloid cells, dendritic cells, lymphocytes, and macrophages identified by its receptors [76]. GM-CSF has mediated cell survival, differentiation, adhesion, growth, and even immune regulation. In mice, GM-CSF k/o mice showed decreases in litter size and litter weights and increased fetal death [77]. GM-CSF is synthesized in the epithelial cells of the female reproductive tract and plays a crucial role by altering its activity during pregnancy [78,79,80,81,82]. GM-CSF appears in its highest levels from conception to early implantation and then gradually declines; then, it is produced by chorionic villi cells and decidual cells until labor [83,84,85]. Unlike most growth factors and cytokines, GM-CSF cannot be produced by the embryo. Rather, as confirmed in both human and mouse models, its receptor synthesis occurs during the preimplantation stage of embryo development [86,87]. Several studies have shown that GM-CSF has positive effects on embryo development, including blastocyst quality, the survival rate, and the expression of genes which are associated with apoptosis [86,88,89]. In other species, mouse embryos cultured with GM-CSF showed evidence of reduced apoptosis [11,90]. There are already commercial embryo culture media supplemented with GM-CSF, namely, EmbryoGen^®^ (Origio, Måløv, Denmark) and BlastGen™(Origio, Måløv, Denmark).

The binding of GM-CSF to its receptor initiates the activation of janus kinase (JAK) 2 and JAK1 kinases, facilitated by the transphosphorylation of these kinases following receptor subunit oligomerization. Activation of JAK kinases leads to tyrosine phosphorylation, creating docking sites for two members of the signal transducers and activators of transcription (STAT) family. GM-CSF functions through the JAK1,2/STAT3,5 pathway, inhibiting the expression of heat shock protein (HSP) and apoptosis pathway genes *Cbl, Hspa5, Hsp90aa1, Hsp90ab1,* and *Gas5*, thereby facilitating the growth and survival of embryos (Figure 1) [89,91]

Of the seven studies involving GM-CSF, six studies were human studies (Table 3). One of these was a human retrospective study of the effect of a dose of 0.6 ng/mL of GM-CSF [92]. The objective of this study was to investigate the effect of relatively low concentrations of GM-CSF on human embryo quality and the clinical outcomes with fresh embryo transfer cycles. This study retrospectively grouped patients by age, based on the use of GM-CSF, and compared them accordingly. Regardless of the patient’s age, there were no significant differences in the cleavage development rate, blastocyst development rate, blastocyst morphology grade, and the clinical outcomes. However, the group with patients aged over 38 years old showed a significant increase in the cleavage development rate (99.4% (GM-CSF) vs. 97.8% (Control), *p* = 0.027) and the blastocyst development rate (45.7% (GM-CSF) vs. 34.9% (Control), *p* = 0.028). There were no comparable data on pregnancy outcomes and infant characteristics. This result demonstrates that 0.6 ng/mL of GM-CSF has no beneficial effect on pregnancy outcomes but has an advantage in patients older than 38 years in terms of increasing the opportunity for embryos.

A study utilizing commercial embryo culture media containing GM-CSF evaluated embryo quality and clinical outcomes among patients meeting inclusion criteria indicative of poor prognoses [93]. The researchers adjusted for age, body mass index, and fertilization procedure for the results. There was no significant difference in the cleavage development rate between the two groups, but there was a significant decrease in blastocyst development rate (OR 0.70, *p* = 0.02) and embryo grade (OR 0.35, *p* = 0.009) in GM-CSF group. After embryo transfer, there was no statistical difference between the two groups in live birth, and the fetal heart rate. In summary, it showed the negative effects of GM-CSF media on the blastocyst development rate and the embryo grade in patients with poor prognoses, while comparable results were observed in clinical outcomes.

Another RCT conducted an explorative secondary analysis based on Ziebe [94] to investigate the implantation potential of poor-quality embryos [95]. The main finding that GM-CSF could not increase the implantation rate for embryos, which are morphologically poor grade on day 3. According to the morphological quality on the day of transfer, there were no statistical differences in total ongoing pregnancy at week 12 and total live birth rate between subjects with GM-CSF and without GM-CSF in top-/intermediate-/poor-grade embryo groups. The researchers concluded that GM-CSF in human embryo culture did not alter clinical results, including ongoing pregnancy rate, live birth rate, and pregnancy loss, in morphological poor-quality embryos. There is a limitation to this study: it shows only favorable effects in top-grade embryo quality and not in the quality of poor-grade embryos.

The earliest human study using GM-CSF containing commercial culture media was carried out with patients who previously failed an ART attempt [96]. Although there was no statistical difference between the two groups in the frequency of the clinical pregnancy rate, the GM-CSF group showed significant increases in the implantation rate at 7 weeks (20.4% (GM-CSF) vs. 11.6% (Control), *p* < 0.05) and 12 weeks (17.4% (GM-CSF) vs. 9.1% (Control), *p* < 0.001). This research demonstrated the positive effects of GM-CSF on the implantation rate of patients who underwent failed IVF/ICSI cycles; however, this study was conducted over a short period of six months, and patient characteristics are lacking.

One of the major studies in which GM-CSF containing culture media could be produced using commercial culture media was conducted with patients from 14 fertility clinics [94]. A total of 1332 patients were mainly divided into two groups: EmbryoAssist medium, with 2 mg/mL human serum albumin (HSA) or 5 mg/mL HSA, and supplementation, with or without 2 ng/mL GM-CSF. There was no statistical difference in the top-quality rate of day-3 embryos. The results showed that supplementation of GM-CSF with low HSA to embryo culture media produced significant increases in the ongoing implantation rate (23.0% (GM-CSF) vs. 19.7% (Control), *p* = 0.02) and the live birth rate (28.9% (GM-CSF) vs. 24.1% (Control), *p* = 0.03). The researchers also showed exploratory data for subgroups who had previous miscarriages. These data showed efficacious results in the ongoing implantation rate (23.2% (GM-CSF) vs. 16.5% (Control), *p* = 0.003) and the live birth rate (29.6% (GM-CSF) vs. 23.1% (Control), *p* = 0.02) when using GM-CSF. This study indicated that GM-CSF had positive effects on the ongoing implantation rate and the live birth rate and that these effects were further enhanced in patients who underwent a previous miscarriage. This study holds significance in demonstrating GM-CSF stability in embryo culture media. Although exploratory subgroup study requires further investigation, this analysis has shown promise that GM-CSF may function positively in particular patients.

Another study determined the developmental potential of early embryos depending on the presence or absence of GM-CSF in embryo culture in a mouse model [97]. The blastomere was mechanically isolated to minimize the specificity of each embryo and was assigned to different groups. The 4-cell rate (59.33% (GM-CSF) vs. 53.57% (Control) *p* < 0.05) showed a significant increase in the GM-CSF group, although no significant difference in blastocyst development rate, blastocyst size (93.89 ± 4.93 µm (GM-CSF) vs. 81 ± 3.02 µm (Control), *p* < 0.05), and cell number (52.8 ± 1.79 (GM-CSF) vs. 44.7 ± 2.84 (Control), *p* < 0.05) were demonstrated in the GM-CSF group.

Based on a multicenter study targeting patients with a history of previous miscarriage, which noted an increase in the ongoing pregnancy rate and live birth rate [94], subsequent RCTs conducted on infertility patient groups have shown either a negative effect [93] or a non-significant effect with GM-CSF [92,95]. There were no consistent research findings demonstrating a positive effect consistent enough to suggest routine use of GM-CSF in specific patient groups. Despite the availability of commercial media containing cytokines with determined optimal concentrations, the widespread adoption of routine application has not ensued. Among the seven RCTs reviewed, three studies indicated a significant increase when GM-CSF was added to the embryo culture medium, while the remaining four studies showed either non-significant or negative effects. This suggests uncertainty regarding the overall positive impact of GM-CSF on a broader scale.

**Table 3:** Effects of GM-CSF in culture media on embryonic development and pregnancy outcomes.

Year	Model	Study Type	Dose of GM-CSF	Timing of Intervention	Cleavage Development Rate	Blastocyst Development Rate	Embryo Grade	Implantation Rate	Live Birth Rate	Ref.
2020	Human (infertility)	Retrospective study	0.6 ng/mL	After fertilization	→,↑	→,↑	→	→	-	[92]
2020	Human (RIF)	RCT	2 ng/mL (EmbryoGen, BlastGen)	COC or oocyte	→	↓	↓	-	→	[93]
2020	Human (infertility)	Explorative secondary RCT	2 ng/mL	COC or oocyte	-	-	-	-	→	[95]
2014	Human (RIF)	RCT	2 ng/mL (EmbryoGen)	COC or oocyte	-	-	→	↑	-	[96]
2013	Human (unexplained infertility)	Multicenter RCT	2 ng/mL	COC or oocyte	→	-	→ day3	↑	↑	[94]
					→	→	→ day3	↑	↑	
2008	Murine	Animal	2 ng/mL	Isolated blastomere from 2-cell stage	↑	→	-	-	-	[97]

-: not analyzed; →: non-significance; ↑: significantly increased; ↓: significantly decreased.

#### 2.2.1. Insulin-like Growth Factor 1 (IGF-Ⅰ)

The human reproductive tract produces numerous growth factors, and the preimplantation embryo expresses those receptors to maintain the maternal–fetal interface. Therefore, clinical reports have been produced concerning the effects of controlled ovarian stimulation using different types of growth factors to increase the success rate IVF [98,99,100,101]. Among various types of growth hormones, the insulin-like growth factor (IGF) family includes peptide hormones consisting of two ligands (IGF-Ⅰ, IGF-Ⅱ), their receptors, and IGF-binding proteins [102,103]. IGF-Ⅰ comprises a small amino acid chain and is linked to its receptor, IGF-ⅠR. Various mammalian species expressed IGF-Ⅰ and IGF-Ⅱ ligands, along with their respective receptors, at the mRNA or protein level in early embryos [6,104,105,106,107]. Additionally, IGF-Ⅰ is produced by the female reproductive tract, the fallopian tube, follicle fluid, and uterine fluid [108,109].

The IGFs are involved in a variety of roles promoting cell growth, proliferation, differentiation, and anti-apoptosis by acting as endocrine, paracrine, and autocrine factors [110,111,112]. IGF plays pivotal roles in various functions, including follicle growth and subsequent embryonic development [113,114,115,116,117]. There have been previous studies utilizing animal models to investigate the effects of IGF in preimplantation embryo culture media; these studies demonstrated an increase in blastocyst formation rate, cell number, glucose uptake, and a decrease in apoptosis [118,119,120]. Additionally, a postnatal experiment in mice indicated a significant reduction in body weight in IGF-Ⅰ(-/-) mutant mice compared to wild-type mice [121].

Growth hormone (GH) and insulin-like growth factor (IGF)—which is closely related to GH—are found in various reproductive tissues, indicating that the GH/IGF axis may directly influence the development. GH and IGF have the ability to modulate key signal transduction pathways involved in cell division and hormone production, such as the mitogen-activated protein kinase (MAPK)/extracellular signal-regulated kinase (ERK), Jak/STAT, and phosphoinositide 3-kinase (PI3K)/protein kinase B (Akt) pathways. When IGF interacts with the insulin receptor, it triggers the recruitment and phosphorylation of either insulin receptor substrate-1 or -2 (IRS-1 or -2). Although IGF-ⅠR and the insulin receptor use the same pathway through IRS-1 and PI3K, IGF-ⅠR acts dependently on IRS-1 and PI3K [122,123]. ERK1/2 enhances cellular mitogenic signals and can be activated by PLC/PKC through increased intracellular calcium and indirectly through PKA. These pathways have downstream associations with S6K1, GSK3, and GLUT4/8, which play a role in apoptosis, glucose homeostasis, glycogen synthase, and glucose transport (Figure 1) [124,125,126,127,128].

The studies related to IGF-Ⅰ were divided into two subcategories: those using a mixture of IGF-Ⅰ with other substances and those using IGF-Ⅰ alone (Table 4). There was a foundational study conducted using IGF-Ⅰ in culture media that are related to the study of human embryos [107], based on which, the effects of IGF-Ⅰ during human preimplantation embryo development were investigated [129]. After fertilization, day 2 embryos of good morphology were cultured with or without 13 ng/mL IGF-Ⅰ up to day 6. Embryos in the IGF-Ⅰ group showed a significantly increased rate of blastocyst formation (74% (IGF-Ⅰ) vs. 49% (Control), *p* < 0.05). In addition, these embryos showed decreased numbers of apoptotic nucleus cells in the IGF-Ⅰ group (16.3 ± 2.9% (IGF-Ⅰ) vs. 8.7 ± 1.5% (Control), *p* < 0.01). However, there was no difference in total cell number between the two groups. The study should have taken into consideration that patient characteristics may not have been randomly distributed and that IGF- I treatment was initiated after day 2. Regardless, this study demonstrated the possibility that IGF-Ⅰ could reduce apoptosis, and outlined the appropriate dose for human embryo culture.

Among the three experiments that examined the impact of using IGF- I exclusively in embryo culture media, the most recent paper examined the effects of IGF-Ⅰ on cat embryo development [130]. This study contained three experimental designs, the last of which experiment was excluded in this review, as it analyzed the effects of IGF-Ⅱ. Using different fertilization techniques, the cleavage development rate was studied. Briefly, one study showed 20 ng/mL of IGF-Ⅰ did not produce a statistically significant change in the cleavage development rate compared to the control group, regardless of fertilization method, though one of the designs showed a significantly higher morula rate (87.0% (IGF-Ⅰ) vs. 52.2% (Control), *p* < 0.05) and blastocyst development rate (43.5% (IGF-Ⅰ) vs. 13.0% (Control), *p* < 0.05) in single-culture group through IVF. In addition, in a group with combined GM-CSF and IGF-Ⅰ, a significant increase only in the morula rate (81.0% (Combination) vs. 52.2% (Control), *p* < 0.05) of the single-culture group was demonstrated, but this did not translate into an increase in the blastocyst development rate. Collectively, the first experiment showed positive effects, the second experiment showed non-significant effects, and the last experiment showed non-significant effects with IGF-Ⅰ. The researchers concluded that the maturation rate increased with IGF-Ⅰ supplementation in the IVM medium; however, caution is required in interpreting this finding as it was inferred from the cleavage development rate.

Another study analyzed the impact of IGF-Ⅰ on the development of mouse embryos [131]. In brief, this study confirmed that regardless of the culture media volume, the presence of 10 ng/mL of IGF-Ⅰ had no impact on the cleavage development rate but notably increased the blastocyst development rate (*p* < 0.05). In addition, this study showed the autocrine role of IGF-Ⅰ by treating embryos with IGF-ⅠR-neutralizing antibodies and demonstrated increased phosphorylation of Akt in blastocysts after a 10 min treatment with 100 ng/mL of IGF-Ⅰ.

**Table 4:** Effects of IGF-Ⅰ in culture media on embryonic development and pregnancy outcomes.

Year	Model	Study Type	Dose of IGF-Ⅰ	Timing of Intervention	Cleavage Development Rate	Blastocyst Development Rate	Implantation Rate	Ref.
2000	Human (infertility)	Prospective study	13 ng/mL	Day 2 embryos of good morphology	→	↑	-	[129]
2021	Cat	Animal	10 ng/mL, 20 ng/mL (GM-CSF 2 ng/mL)	COC	→	→, ↑ (20 ng/mL)	-	[130]
			20 ng/mL (IGF-Ⅱ 20 ng/mL)	COC	→	→	-	
			20 ng/mL	Oocyte	→	-	-	
					→	-	-	
2019	Yak-cattle crossbred	Animal	100 ng/mL (EGF 10 ng/mL, L-cysteine 0.6 mM)	COC	-	↑	-	[132]
2013	Murine	Animal	10 ng/mL	Zygote	→	↑	-	[131]
2013	Bovine	Animal	10 ng/mL (EGF 10 ng/mL)	Zygote	→	↑	-	[133]
2010	Bovine	Animal	500,000 ng/mL (IGF-Ⅱ 10 µg/mL, bFGF 25 µg/mL, TGF- β1 1 µg/mL, GM-CSF 1 µg/mL, LIF µg/mL)	Zygote	-	↑	-	[134]
2003	Bovine	Animal	50 ng/mL	Zygote	→	↑	-	[120]

-: not analyzed; →: non-significance; ↑: significantly increased.

Finally, a study determined the effects of IGF-Ⅰ and epidermal growth factor (EGF) in bovine embryo culture medium, respectively; however, we specifically emphasized the role of IGF-Ⅰ, as the combination outcomes were identical to the outcomes when each substance was used individually [120]. In the experimental results for IGF-Ⅰ, this study showed significant differences in the blastocyst development rate (56.1% (IGF-Ⅰ) vs. 43.4% (Control), *p* < 0.01), the total cell number (162 ± 6.83 (IGF-Ⅰ) vs. 141 ± 6.59 (Control), *p* < 0.05), and the apoptosis rate (2.1 ± 0.28 (IGF-Ⅰ) vs. 3.3 ± 0.42 (Control), *p* < 0.05) between the two groups. This study provided support for the positive effects of IGF-Ⅰ on in vitro bovine blastocyst development, particularly in reducing apoptosis.

#### 2.2.2. Insulin-like Growth Factor 1 (IGF-Ⅰ) Combined with Other Supplements

There are three studies that investigated the results of treating embryo culture media with a combination of IGF-Ⅰ and other substances such as EGF, GM-CSF, IGF-Ⅱ, etc.

The substances of IGF-Ⅰ, cysteine, and EGF treated with yak–cattle crossbred embryos before fertilization [132]. All the parameters showed positive results in the maturation rate (84.44 ± 0.02 (Combination) vs. 66.50 ± 0.04 (Control), *p* < 0.05), the cleavage development rate (80.45 ± 0.12 (Combination) vs. 64.77 ± 0.10 (Control), *p* < 0.05), and the blastocyst development rate (38.67 ± 0.06 (Combination) vs. 21.16 ± 0.08 (Control), *p* < 0.05) with the combination group compared to the control group. In addition, with treated IGF-Ⅰ alone, there was an increase in the blastocyst development rate (30.06 ± 0.05 (IGF-Ⅰ) vs. 21.16 ± 0.08 (Control), *p* < 0.05), but the cleavage development rate did not show a statistical difference. The researchers concluded that the combination of IGF-Ⅰ, cysteine, and EGF could enhance maturation rate and embryo development.

In another study, using bovine embryos, the effects of the combination of IGF-Ⅰ and EGF on embryo culture media were investigated [133]. There was a non-significant difference in the cleavage development rate between the groups, while the blastocyst development rate was significantly increased in IGF-Ⅰ with the EGF group (23.5% (Combination) vs. 17.1% (Control), *p* < 0.05) and the EGF-alone group. In addition, the apoptosis rate in blastocysts showed statically decreased data in the IGF-Ⅰ with EGF (10.5 ± 0.7 (Combination) vs. 21.9 ± 1.6 (Control), *p* < 0.05) group and EGF-alone group compared to the control group, respectively.

There is another study on the impact of a combination of various growth factors and cytokines on bovine embryo culture medium [134]. The culture medium contained six kinds of supplements: IGF-Ⅰ, IGF-Ⅱ, transforming growth factor beta (TGF-β), GM-CSF, basic fibroblast growth factor (bFGF), and LIF. The researchers obtained positive results regarding the effects of supplements on the blastocyst development rate (45% (Combination) vs. 24% (Control without serum), *p* < 0.05) and the blastocyst cell number (154 ± 15 (Combination) vs. 125 ± 11 (Control without serum), *p* < 0.05). Combination groups showed a significant increase in hatched blastocyst development rate, compared to the control, with 10% fetal calf serum (72% (Combination) vs. 48% (Control with serum), *p* < 0.05). This study demonstrated that growth factors and cytokines could act synergistically to promote embryo development competency, especially in blastocysts; nevertheless, accurate data on the viability and quality of embryos may require additional assessment.

Overall, the lack of implantation data in IGF-Ⅰ studies limits the precise analysis of the effects when IGF-Ⅰ is added to embryo culture media. Despite the addition of combination supplementations, all studies consistently show a non-significant result in the cleavage development rate [120,129,130,133]. However, most of the research results, including the study that solely added IGF-Ⅰ, showed an increase in the blastocyst development rate [120,129,130,132,133]. There are limited data on the effects of adding IGF-Ⅰ to embryo culture media in terms of obtained clinical outcomes, both in animal models and in human models. Most of the studies examining the effects of IGF-Ⅰ supplementation involve its application in combination with other supplementations rather than alone. Further research is needed to investigate the effects of adding IGF-Ⅰ alone to embryo culture media. The routine application of IGF-Ⅰ in embryo culture media would require human clinic data and studies that examine pregnancy outcomes to support its effectiveness.

#### 2.2.3. Leukemia Inhibitory Factor (LIF)

Leukemia inhibitory factor (LIF), an interleukin 6 class cytokine, is expressed in the trophectoderm and regulates various cellular functions by binding to specific receptors, LIFR, and glycoprotein 130 [135]. These receptors have two forms: membrane-bound and soluble [136]. LIF contributes to cellular growth, proliferation, and survival through cell signaling pathways.

In the various cell types, LIF could promote JAK/STAT, PI3K/PKB, and ERK/MAPK pathways [137,138,139,140,141]. Within a mouse model study, the LIF signaling mechanism in uterine tissue and trophoblast cells occurs through the activation of the JAK1/STAT3 pathway [142,143]. LIF promotes receptor heterodimerization, leading to the activation of a JAK/STAT pathway. JAK1 phosphorylates tyrosine residues, providing binding sites for STAT3. Multiple studies have shown that STAT3 stimulates various downstream genes related to proliferation, angiogenesis, differentiation, cell cycle progression, and apoptosis, such as *c-Myc*, *VEGF*, *HIF1a*, *Rac1*, *Notch1*, *p53*, and *Bcl-2* (Figure 1) [144,145,146,147,148,149,150,151,152,153,154,155].

It has been observed that human oocytes, cleavage, and blastocysts express LIFR and gp130 in mRNA and protein levels [156]. These receptors, expressed in the endometrium and upregulated during the luteal phase coinciding with implantation, correlate with studies associating LIF with the implantation process [157,158,159,160]. In a mouse study, gp130 k/o and LIFR k/o types of embryos could not survive during post-implantation development [161]. Indeed, LIF has been extensively studied and found to play a crucial role, particularly during the process of implantation in pregnancy in various species [162,163]. Another study showed that supplements of LIF could increase the cell number in mouse blastocysts and in gene expression of *Oct-4* and *Cdx22*, related to ICM and trophectoderm, respectively [164]. Through various human and animal studies, it has been demonstrated that LIF plays a crucial role in not only implantation but also embryo development [165,166]. There is a study that higher concentration of LIF in embryo culture media is associated with a higher pregnancy rate when blastocysts are transferred [167]. Recently, the expression of LIF in follicular fluid and ovarian stromal cells has also been associated with young women with poor ovarian response during ovarian stimulation cycle [168].

Six studies have analyzed the effects on embryonic development of adding LIF to embryo culture media (Table 5). Among them, three studies focused on human models, while the remaining three studies investigated animal models. Furthermore, four studies specifically investigated the effects of adding LIF alone to the culture medium, while the remaining two studies assessed the use of a combination of LIF with other supplements.

A RCT with human frozen–thawed cleavage was conducted in early 2000 to evaluate the effects of LIF [169]. It used two kinds of base culture media, human tubal fluid medium, and M3 medium. The results showed no significant difference in cleavage development rate with the addition of LIF. However, there was a significant increase in both morula rates using HTF medium for the base medium (23.2% (LIF) vs. 6.9% (Control-1), *p* < 0.05) (23.1% (LIF) vs. 19.7% (Control-2), NS) and the blastocyst development rate (11.0%, 12.8% (LIF) vs. 0% (Control), *p* > 0.05). The researchers concluded that LIF has the potential to affect early embryo development, but further, larger-scale multicenter studies are needed to confirm these findings.

Another study was performed to assess blastocyst development, total cell number, hatching rate, and LIF and its receptor expression in preimplantation buffalo embryos [170]. The group of LIF supplements demonstrated a positive influence on blastocyst development. There was no significant difference in the cleavage development rate and the morula rate. In terms of blastocyst development parameters, the expanding blastocyst development rate (16.57 ± 1.13 (LIF) vs. 9.80 ± 1.19 (Control), *p* < 0.05) and total cell number (81.5 ± 2.93 (LIF) vs. 70.5 ± 2.2 (Control), *p* < 0.05) were significantly increased in the LIF group. Additionally, the expression of LIFR mRNA and protein was confirmed from the oocyte to blastocyst stages, while the expression of LIF ligand mRNA and protein was observed from the 8 cell stage to the blastocyst stage.

**Table 5:** Effects of LIF in culture media on embryonic development and pregnancy outcomes.

Year	Model	Study Type	Dose of LIF	Timing of Intervention	Cleavage Development Rate	Blastocyst Development Rate	Embryo Grade	Implantation Rate	Live Birth Rate	Ref.
2019	Human (infertility)	RCT	5 ng/mL (GM-CSF 2 ng/mL, HB-EGF 5 ng/mL)	After ICSI	→	↑	→, ↑ day 3, day 5/6	↑, → Fresh, FER	↑	[171]
2000	Human (infertility)	RCT	1000 IU/mL	Cleavage after vitrified-thawed	→	↑	-	-	-	[169]
2021	Bovine	Animal	20 ng/mL (FGF2 40 ng/mL, IGF-Ⅰ 20 ng/mL)	Putative zygote	→	↑	-	-	-	[172]
2013	Buffalo	Animal	100 ng/mL	Presumptive zygote	→	↑	-	-	-	[170]
2008	Murine	Animal	1500 IU/mL	Isolated blastomere from 2-cell stage	↑	→	-	-	-	[97]

-: not analyzed; →: non-significance; ↑: significantly increased.

In the previously mentioned paper on GM-CSF, the researchers also investigated the impact of supplementing the embryo culture medium with LIF on mouse embryo development [97]. Using isolated blastomeres from mouse 2 cell embryos, LIF showed a significant increase in cleavage development rate (61.10% (LIF) vs. 52.4% (Control), *p* < 0.001) after 48 h of culture. There was no significant difference in the blastocyst development rate between the two groups, but the total cell number (53.4 ± 1.82 (LIF) vs. 44.7 ± 2.84 (Control), *p* < 0.05) was higher in the LIF group.

#### 2.2.4. Leukemia Inhibitory Factor (LIF) Combined with Other Supplements

Two studies used LIF with other supplementations such as GM-CSF, HB-EGF, FGF2, and IGF-Ⅰ. An RCT was conducted to investigate the effects of culture media integrated with LIF, GM-CSF, and heparin-binding epidermal growth factor-like growth factor (HB-EGF) on embryonic development and pregnancy outcomes with a human ICSI cycle [171]. The incorporation of cytokines and growth factors could have directly targeted potential effects in hatching or implantation, leading to improved clinical outcomes for the recurrent implantation failure (RIF) and pregnancy-loss groups. The cytokines and growth factors demonstrated potential effects on clinical outcomes in terms of ongoing pregnancy rate (47% (Combination) vs. 36% (Control), *p* = 0.012) and live birth rate (45% (Combination) vs. 33% (Control), *p* = 0.0062). In addition, embryologic characteristics and blastocyst development rate (68% (Combination) vs. 61% (Control), *p* = 0.0001), showed a good quality of blastocyst development rate according to Istanbul guidelines (48% (Combination) vs. 36% (Control), *p* = 0.0001), and implanted fresh embryo rate (38% (Combination) vs. 30% (Control), *p* = 0.023) showed a statistical increase in the combination group while cleavage development rate was statistically non-significant. This study showed that the integration of LIF, GM-CSF, and HB-EGF could enhance embryonic development and pregnancy outcomes with no adverse consequences.

The remaining studies all focused on the effects of supplementation in mammalian embryos, primarily observing the development of embryos without pregnancy outcomes. In 2021, the effects of LIF, IGF-Ⅰ, and fibroblast growth factor 2 (FGF2) on bovine embryo development were studied [172]. Although there was no significant difference in cleavage development rate between the supplementation group and control group, the supplementation group exhibited an increase in blastocyst development rate (46.2 ± 1.3 (Combination) vs. 32.9 ± 1.3 (Control), *p* < 0.05). However, there were no statistical differences in total blastocyst cell number. After the blastocyst was frozen–thawed, the hatching rate (40.4% ± 0.04 (Combination) vs. 19.8% ± 0.04 (Control), *p* < 0.05) and apoptosis index (6.70% ± 1.32 (Combination) vs. 18.06% ± 1.62 (Control), *p* < 0.05) was statistically significantly different between the two groups. This study suggests that LIF supplementations have positive influences on blastocyst development rate and its quality.

These two studies both showed an increase in blastocyst development rate although they used different combinations of supplementations and models. Both studies selected supplementations based on other research that demonstrated their positive effects [134,173].

In conclusion, LIF supplementation of embryo culture media showed developmental potential in blastocysts. Only one human RCT has demonstrated positive effects in implantation and birth outcomes, although they used a mixture of supplementations [171]. All of the studies showed an increase in the blastocyst development rate using LIF or a LIF combination [169,170,171,172], except for one study, which showed no significant effect [97]. While the diverse roles and significant importance of LIF in embryo development and the pregnancy process have been well established, there have been relatively few studies on the supplementation of LIF in embryo culture media. Therefore, there may need to be more studies including pregnancy outcomes and human RCT.

## 3. Conclusions

In this review, we summarized the effects on embryonic development of several representative supplements added to culture media. Melatonin, GM-CSF, IGF-Ⅰ, and LIF all showed positive effects on embryo-related outcomes such as blastocyst development rate and the grade of the embryo. Melatonin, GM-CSF, IGF-Ⅰ, and LIF seem to have a generally positive effect on pregnancy-related outcomes such as the implantation rate and the live birth rate. Studies have been recently published on the effect of supplements in different clinical situations, such as old age, RIF, and unexplained infertility. In the case of melatonin and GM-CSF, many clinical studies have been conducted on humans, but for the rest of the supplements, only preclinical studies on murine and bovine models have been conducted. The results of human studies should be built on with future clinical trials. In addition, studies to find the optimal dose and timing of intervention should be continued, as each study used different doses of supplements and timings of intervention. Supplementation strategies in culture media have shown promise in enhancing blastocyst development rates and improving certain aspects of ART outcomes. However, the translation of these benefits into increased live birth rates requires further investigation. Robust clinical studies encompassing a wider range of substances and participant profiles are essential to unraveling the true potential of supplementation in culture media. Ultimately, understanding the molecular mechanisms behind these effects will enable us to use an embryo culture medium containing personalized supplements tailored to the individual circumstances of infertility patients, bringing renewed hope to those on the journey to parenthood.

## Figures and Tables

**Table 1:** Components of single-step media for human embryo culture and their concentrations.

Component	Role in Embryo Development	Global-Total	CSC	G-TL	1-Step
Glucose (mM)	Primary energy source during postcompaction stage	0.18	0.47	0.97	0.19
Lactate (mM)	Primary energy source during precompaction stage	4.9	5.71	10.01	4.35
Pyruvate (mM)		0.24	0.28	0.55	0.22
Essential amino acids (μM)					
Arg	Osmolytes	278	292	324	336
Cys	Buffers of internal pH	32	34	26	28
His		76	80	89	90
Ile	Antioxidants	182	199	215	204
Leu	Protein synthesis, antioxidants	177	188	204	206
Lys	Chelators for heavy metals	154	168	182	182
Met	Osmolytes, chelators for heavy metals	44	50	54	54
Phe	Antioxidants	79	83	91	92
Thr	Energy source, osmolytes, antioxidants	162	176	184	204
Trp	Biosynthetic precursor molecules	18	20	21	23
Tyr	Protein synthesis	69	75	80	83
Val	Biosynthetic precursor molecules, antioxidants	163	174	200	196
Nonessential amino acids (μM)					
Ala	Buffers of internal pH, antioxidants	46	48	63	38
Asn	Energy source	42	46	40	36
Asp	Energy source, osmolytes, chelators for heavy metals	42	43	12	58
Glu	Energy source, osmolytes	40	41	0	49
Gln	Energy source, biosynthetic precursor molecules, protein synthesis	0	0	10	36
Gly	Energy source, osmolytes	42	44	185	48
Pro	Osmolytes	55	60	126	66
Ser	Osmolytes, antioxidants	40	42	96	46
Tau	Antioxidants	0	0	48	0
Calcium (mM)	Metabolic parameters and macromolecular synthesis through cell-to-cell interaction	1.6	1.9	1.0	2.1
Magnesium (mM)		0.24	0.78	1.62	1.78
Potassium (mM)	High concentrations in oviduct fluid relative to serum	2.8	2.8	5.5	2.9

## Data Availability

Not applicable.

## Abbreviations

IVF	in vitro fertilization
ART	assisted reproductive technology
Nrf2/ARE	nuclear factor erythroid 2-related factor 2/antioxidant-responsive element
VEGF	vascular endothelial growth factor
ATP	adenosine triphosphate
RCT	randomized clinical trial
hCG	human chorionic gonadotropin
DNA	deoxyribo nucleic acid
PCOS	polycystic ovary syndrome
IVM	in vitro maturation
GM-CSF	granulocyte–macrophage colony-stimulating factor
HSP	heat shock protein
GH	growth hormone
IGF	insulin-like growth factor
MAPK	mitogen-activated protein kinase
ERK	extracellular signal-regulated kinase
IRS	1insulin receptor substrate
EGF	epidermal growth factor
bFGF	basic fibroblast growth factor
LIF	leukemia inhibitory factor
JAK	Janus family tyrosine kinase
STAT	signal transducers and activators of transcription
PI3K	phosphoinositide 3-kinase
Akt	protein kinase B
ROS	reactive oxygen species
GPCR	G protein-coupled receptor
O^2−^	superoxide anion
H_2_O_2_	hydrogen peroxide
•OH	hydroxyl radical
SOD	superoxide dismutase
GSH	glutathione peroxidase
Th1	Type 1 helper T cells
Th2	Type 2 helper T cells
HSA	human serum albumin
HB-EGF	heparin-binding epidermal growth factor-like growth factor
RIF	recurrent implantation failure
FGF2	fibroblast growth factor 2
